# Mr. Hume, on the Cupping Glass in Hydrophobia

**Published:** 1804-12-01

**Authors:** Jos. Hume

**Affiliations:** Long Acre


### *To the Editors of the Medical and Physical Journal.*

Gentlemen,

It is incumbent on me to observe, that the air-pump water-bath, although unknown to me, had certainly been suggested to *remove poisons* from the surface of the body, before I sent in my last communication; I, therefore, claim no more merit respecting the mere hint which I laid before you, than this, that, where a common *air-pump* is at hand, any other kind of vessel, furnished (in a manner that needs no description) with a proper *stop-cock*, may possibly prove always successful, if *instantly* applied.

To do justice to the author, I shall quote the following passage from the work itself.

"It may not a little surprise some of my readers, that I have not expatiated on the efficacy of the apparatus in the removal of animal, vegetable, and mineral poisons recently applied to the system; especially, as the extraction of of poison from a wound originally gave rise to the invention." (See Dr. Blegborough on the air-pump vapour-bath, p.136.)

The whole of my last short, and, I confess, very hastily written letter, was composed in a *conditional* sense. Nothing in it amounts to an assertion; the whole was meant to be, and, I am confident, will be found to be, merely *recommendatory*; I cannot, therefore, either retract a single syllable, or answer any *public* question respecting its contents.

I know my letter has been most miserably garbled. A studied exordium with "*no errors should be permitted to pass unnoticed; the wide ocean of medical science; the dangerous rocks; wild and extravagant fancies; speculators in physic, &c.*" must prepare the reader to give credit to the author, and to follow him implicitly, if nothing be questioned respecting the text.

I have not the least doubt but that whoever has read the comment upon my letter, must suppose that I expect complete success from the application of a cupping-glass, even when the *barking, biting, anxiety, fever,* and *dread* of *swallowing* are hastening the patient to his grave. This would indeed be an *immediate cure.*

The commentator must have seen what I meant by the general tenor of the whole letter, besides this sentence, "*the moment* an accident of this kind has happened is *evidently* the only period to apply the glasses with *any prospect of success.*"

The patient *cured* by the commentator does not appear to have been diseased, or the puny history must be defective. It does not appear that the *pointer* dog was really capable of communicating *hydrophobia,* for the animal, with all the *unequivocal* symptoms, did not die *of the disease*; perhaps the poor creature was shot, or hanged and quartered, and then can tell no tales.

Amongst some other remarks, which deserve to be studied, I observe in the same number of the Medical and Physical Journal, p. 387—391, that Professor Rossi was deceived, and why not also our commentator? Another may also be seen, of two examples of "persons dying under *hydrophobic* symptoms from *biting their own fingers,* in a paroxsysm of *anger!* How opportunely these observations occur in the self same Journal; they appear not only as a *preventive* but as an *immediate cure.*

I observe some singular opinions respecting the *double* effect of alkali, which I could not comprehend, but on which which one might have said more than would be palatable; perhaps animal poisons are *acids*: if so, here is a new field opened to the chemist, if he be not afraid to "turn his mind to surgery." But, "*ne ultra crepidam.*"

I have considered, that to contend with a competitor so powerful would be madness. What chance could I expect, to wrestle with a physical Hercules? with, not only a member of the Royal College of Surgeons, London, but who can add the strength of an apothecary, of a man-midwife, and of a vendor of drugs, chemicals, &c. to whatever besides he may desire to possess!

The compliment paid to me on some observations, which appeared in your Journal for May and July, I cannot accept—the donor can be no very perfect judge of *nomenclature*, who confounds *calx cum kali puro* with what is *commonly called lapis infernalis*. How far this accords with "preparing prescriptions with accuracy," I leave to the judgement of others.

I am, &c.

*Long Acre, Nov.* 14, 1804. JOS. HUME.

---

